# Genetic Association of Transcription Factor 7-Like-2 rs7903146 Polymorphism With Type 2 Diabetes Mellitus

**DOI:** 10.7759/cureus.52709

**Published:** 2024-01-22

**Authors:** Santosh Kumar, Pritam Prakash, Rekha Kumari, Naresh Kumar

**Affiliations:** 1 Biochemistry, Indira Gandhi Institute of Medical Sciences, Patna, IND; 2 Medicine, Indira Gandhi Institute of Medical Sciences, Patna, IND

**Keywords:** allele-specific pcr, t2dm, rs7903146, tcf7l2, single nucleotide polymorphism

## Abstract

Introduction: Type 2 diabetes mellitus (T2DM) mainly results from the inability of muscle, fat, and liver cells to uptake glucose due to insulin resistance or deficiency of insulin production by the pancreas. Predisposition to T2DM may be due to environmental, hereditary, or both factors. Although there are many genes involved in causing T2DM, transcription factor 7-like-2 gene (TCF7L2) rs7903146 (C/T) single nucleotide polymorphism (SNP) found in genome-wide association studies (GWAS) is susceptible to T2DM. TCF7L2 is involved in pancreatic beta cell proliferation and differentiation via the Wnt signaling mechanism.

Objectives: To find the genetic association of TCF7L2 rs7903146 (C/T) gene polymorphism in patients with T2DM.

Methods: A case-control study was conducted on 194 T2DM patients recruited from the endocrinology department at Indira Gandhi Institute of Medical Sciences, Patna, and 180 non-diabetic healthy controls that were age and sex-matched with the patients. All clinical examination and biochemical investigations like glycosylated hemoglobin (HbA1c), total cholesterol, triglycerides, high-density lipoprotein-cholesterol, and low-density lipoprotein-cholesterol; and determination of TCF7L2 gene polymorphism by allele-specific polymerase chain reaction (AS-PCR) were carried out for each subject.

Results: The T allele of the rs7903146 (C/T) SNP was associated with a two-fold higher risk of T2DM and the heterozygous genotype (CT) with a 1.96 times higher risk.

Conclusion: There is a high association of this SNP with the development of T2DM in the eastern Indian population. Serial monitoring of HbA1c should be done in an individual having this type of polymorphism for early detection of T2DM to prevent future complications.

## Introduction

Diabetes is a chronic, metabolic disease characterized by elevated levels of blood glucose (or blood sugar), which leads over time to serious damage to the heart, blood vessels, eyes, kidneys, and nerves. The most common is type 2 diabetes, usually in adults, which occurs when the body becomes resistant to insulin or doesn’t make enough insulin [1]. In recent years, the prevalence of diabetes in India has increased. According to the data from the National Noncommunicable Disease Monitoring Survey, the prevalence of diabetes in India was 9.3% in 2022 [2].

TCF7L2 is a nuclear receptor for CTNNB1 (B catenin) that is involved in the Wnt signaling pathway which regulates the secretion of glucagon-like peptide-1 (GLP-1) produced by intestinal endocrine L cells. Alteration in the Wnt pathway leads to reduced GLP-1 secretion which affects the insulin secretion and generation of B cells. TCF7L2 is also involved in the activation of mRNA expression of glucagon and GLP-1 in the gut endocrine cell. It stimulates insulin secretion, inhibits glucagon, and increases insulin sensitivity. Therefore, any defect in TCF7L2 predisposes the subject to type 2 diabetes mellitus (T2DM) [3].

The TCF7L2 gene is located on the long arm of chromosome 10. The single nucleotide polymorphism (SNP) rs 7903146 is situated in the intronic region of the gene. TCF7L2 gene produces a transcription factor that is important for the Wnt signaling pathway, and mutant alleles appear to be associated with dysfunction in insulin secretion/beta-cell function [4].

More than 20 genes and their variants have been shown to be associated with diabetes out of which transcription factor 7-like-2 (TCF7L2) rs7903146 (C/T) SNP gene polymorphisms have been frequently involved with T2DM [5].

In 2006, TCF7L2 gene polymorphisms were identified in the Icelandic population in a binding region of chromosome 10 that were strongly associated with the risk of type 2 diabetes [6]. Soon this association was found in several ethnic groups, with an allele relative risk of 1.4. This is the strongest association observed among the genes generally associated with type 2 diabetes susceptibility [7].

The present study aimed to find the distribution and association of risk alleles for TCF7L2 rs7903146 (C/T) SNP in the eastern population of India with T2DM.

## Materials and methods

Study design

It was a cross-sectional observational study conducted in the Department of Biochemistry at Indira Gandhi Institute of Medical Sciences (IGIMS), Patna to know the association of TCF7L2 gene variants in type 2 diabetes patients in the Indian population. The study period was two years and a half years. Appropriate informed consent was taken from all study subjects. The Institutional Ethics Committee, IGIMS, Patna issued approval 63/IEC/IGIMS/2021.

Diagnosed cases of T2DM according to criteria laid by the American Diabetes Association (ADA) 2020 having ages between 30-80 years were included in the case group. Patients not willing to participate in the study and pregnant females were excluded from the study.

Sample size

The case group had 194 patients diagnosed with T2DM. In all 180 healthy volunteers, having age and sex matched with the patient group, were recruited in the control group.

Sample collection

For plasma glucose estimation, 2 ml of fasting (fasting at least 8 h) and 2 ml of post-prandial blood samples were drawn into fluoride tubes from all participants. Another 3 ml of blood was drawn in a plain vacutainer tube and was used to analyze the biochemical parameters in serum. Apart from that a 2 ml of blood sample was taken in an ethylenediaminetetraacetic acid (EDTA) tube for estimation of glycosylated hemoglobin (HbA1C).

SNP analysis

For each subject, DNA was extracted using a QiAmp Blood DNA extraction kit (Qiagen, USA) out of which 200µl blood was taken from the sample drawn for HbA1C estimation prior to centrifugation. The absorption ratio A 260/280 and A 260/230 of the extracted DNA was measured using a Nanodrop spectrophotometer. The quantity of extracted DNA was adjusted to a final concentration of 30 ng/µl suitable for optimized polymerase chain reaction (PCR) protocol. Allele-specific PCR for SNP rs 7903146 located in the TCF7L2 gene was carried out with the concentration-adjusted DNA sample. The 25 µl PCR reaction mix consists of Go Taq PCR mix (Promega, USA) 12.5µl, 0.5µl of common reverse primer, 0.5µl of either of the allele-specific primer mixed with 2.5 µl template DNA, and 9 µl water. The cycling parameter consists of initial denaturation at 95^o^C for 8 min followed by 30 cycles of denaturation at 95^o^C for 45 sec, annealing at 52^o^C for 1 min, and extension at 72^o^C for 40 sec. The final extension step was carried out at 72^o^C for 5 min. For each subject, there were two separate PCR reactions with a combination of either C or T allele-specific primer but the same common reverse primer (Table 1).

**Table 1:** Sequence of primers used in the AS-PCR reaction and sequencing reactions. AS-PCR: Allele-specific polymerase chain reaction.

Primers	Primer sequence 5’ -3’
rs7903146 C	GAACAATTAGAGAGCTAAGCACTTTTTAGAGAC
rs7903146 T	GAACAATTAGAGAGCTAAGCACTTTTTAGAGAT
Common reverse primer	AGATGAAATGTAGCAGTGAAGTGC
Sequencing primer TCF7L2_F	ATGGTGACAAATTCATGGGC
Sequencing primer TCF7L2_R	AGATGAAATGTAGCAGTGAAGTG

**Figure 1:**
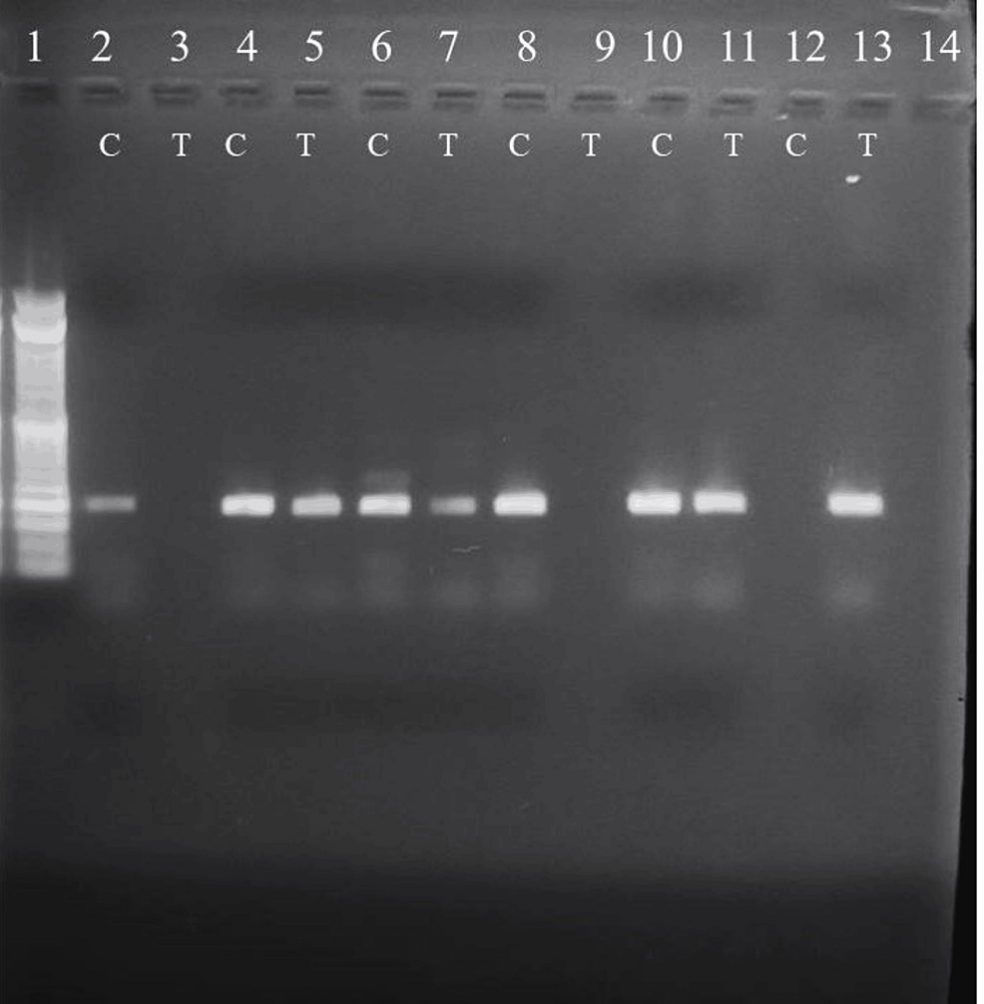
Gel showing the amplified PCR product of T2DM subjects. Lane 1: 50bp ladder. Lanes 2,3; 8,9: homozygous CC. Lanes 4,5;  6,7; 10,11: heterozygous CT. Lanes 12,13 C&T pair: homozygous TT. PCR: Polymerase chain reaction; T2DM: Type 2 diabetes mellitus.

**Figure 2:**
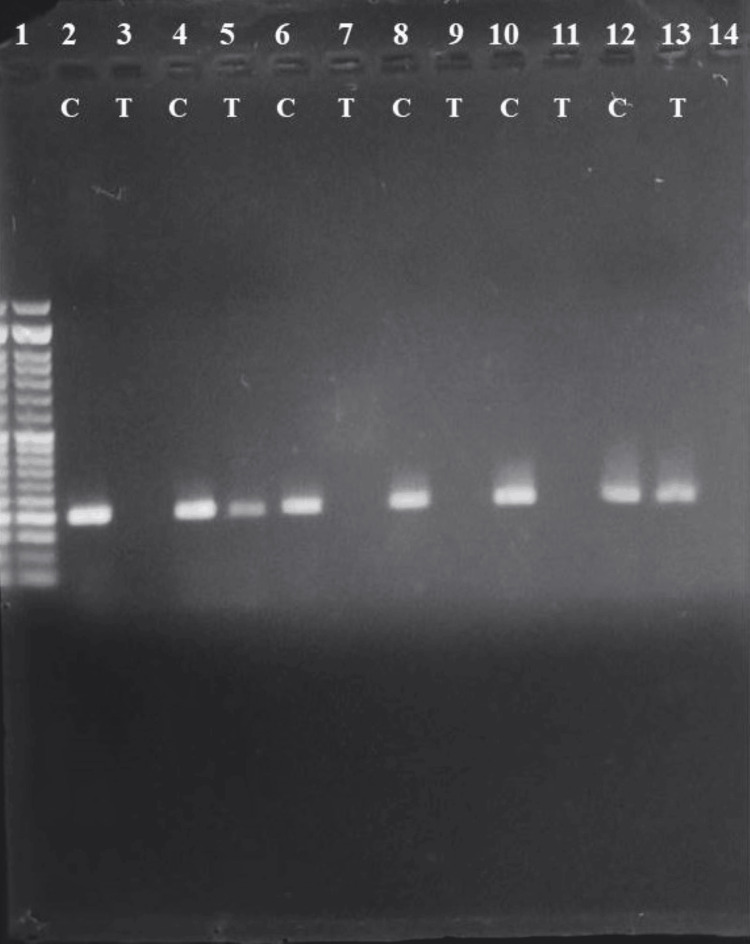
Gel showing the amplified PCR product of control subjects. Lane 1: 50bp ladder. Lanes 2,3; 6,7; 8,9; 10,11: homozygous CC. Lanes 4,5; 12,13 C&T pair: heterozygous CT. PCR: Polymerase chain reaction.

The amplified PCR product of these two reactions was run in separate adjacent lanes for each subject and analyzed in 2% agarose gel stained with ethidium bromide under a UV light trans illuminator. The appearance of the band in the lane loaded with C primer PCR reaction product indicates the presence of C nucleotide at the SNP site and likewise, the presence of T nucleotide at the SNP site can be inferred by observing the band in the lane with T primer PCR reaction product (Figures 1, 2).

Estimation of biochemical parameters

Low-density lipoprotein (LDL) cholesterol, high-density lipoprotein (HDL) cholesterol, triglyceride, total cholesterol, HbA1C, and fasting and post-prandial plasma glucose levels were measured on AU 5800 autoanalyzer (Beckman and Coulter, USA).

Statistical analysis

Statistical analysis was performed using Statistical Package for Social Sciences (SPSS), version 16.0 (SPSS Inc., Chicago, USA). The Shapiro-Wilk test was used to test the normality of the data. Categorical variables were expressed as frequencies and percentages and continuous variables as medians and interquartile ranges (IQR). Allele and genotype frequencies were compared using Fisher’s exact test. Differences in clinical and biological parameters were compared between groups by non-parametric Mann-Whitney test. The significance level was set at 5%.

## Results

The mean age of the T2DM subjects was 54.5 ± 10.7 years and that of the control group was 44.68 ±10.9 years. There were 129 males and 65 females who were diabetic and 95 males and 85 females were in the control group.

In the diabetic group, all parameters, viz. HbA1c, glucose (fasting, post-prandial, and random), LDL, very low-density lipoprotein (VLDL), total cholesterol, and triglycerides were increased highly significantly. Moreover, a significant decrease in serum HDL cholesterol was observed in the patient group (p < 0.05) compared to the control group (Table 2).

**Table 2:** Comparison of various parameters in case and control group. HbA1c: Glycosylated hemoglobin; HDL: High-density lipoprotein; IQR: Interquartile range; LDL: Low-density lipoprotein; VLDL: Very low-density lipoprotein; *Mann Whitney test.

Test parameters	Cases (n=194) Median (IQR)	Control (n=180) Median (IQR)	p-value*
HbA1c (%)	7.45(2.2)	5.1(0.5)	< 0.05
Fasting plasma glucose (mg/dl)	145(62.5)	102(22)	< 0.05
Post-prandial plasma glucose (mg/dl)	247(122.5)	130(11)	< 0.05
Random blood glucose (mg/dl)	198(71.5)	126.5(15)	< 0.05
LDL cholesterol (mg/dl)	113(53)	66(24)	< 0.05
HDL cholesterol (mg/dl)	39(10.25)	44.5(11)	< 0.05
VLDL cholesterol (mg/dl)	29(15.25)	24(8)	< 0.05
Triglyceride (mg/dl)	121(83)	76(24.75)	< 0.05
Total cholesterol (mg/dl)	177(57.25)	124.5(26.75)	< 0.05

The TCF7L2 was genotyped in all studied subjects; the descriptive and comparative statistics of the genotype frequencies of the TCF7L2 rs7903146 (C/T) polymorphism are illustrated in (Table 3). In diabetic patients, 24.2% had the wild-type CC genotype, 39.3% had the heterozygous CT genotype, and 16.5% had the mutant TT genotype. On the other hand, 38.9% of the controls had the CC genotype, 48.3% had the CT genotype, and 12.8% had the TT genotype. There were statistically significant differences observed between the cases and controls regarding the genotype frequencies (p< 0.05).

**Table 3:** Comparison between genotype and allele frequencies of rs7903146 TCF7L2 (C/T) polymorphism in case and control group. *Fisher’s exact test.

Genotype	Cases (n=194)	Control (n=180)	OR (95% CI)	p-value*
TT (mutant)	32	23	2.072	0.027
CC (wild)	47	70		
CT (heterozygous)	115	87	1.969	0.004
CC (wild)	47	70		
Allele				
T	179	133	1.462	0.011
C	209	227		

As for the allele frequencies, descriptive and comparative statistics in Table 3 show that the C allele was present in 53.9% of the cases and 63.1% of the controls (p<0.05). The T allele was found in 46.1% of the cases and 36.9% of the controls (p < 0.05). There was almost a two-fold risk associated with the mutant homozygous TT genotype as compared to the wild-type CC genotype. A similar risk of 1.96 times was associated with heterozygote CT compared to CC.

**Figure 3:**
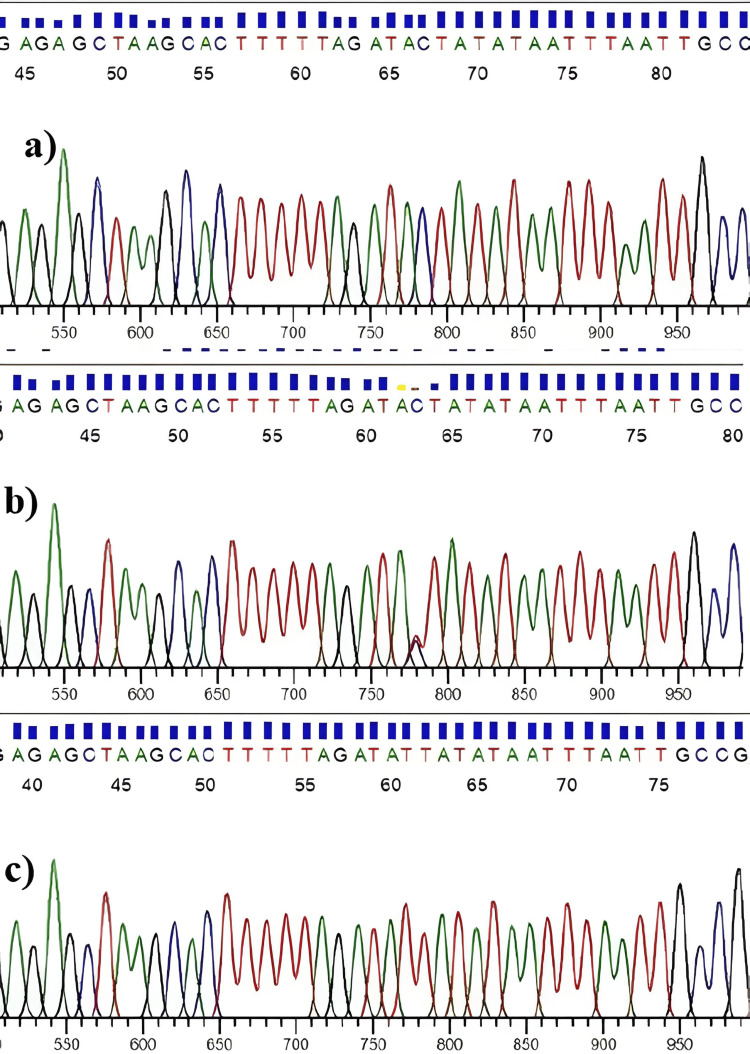
Sequencing result of the amplified PCR product having the SNP site using sequencing primer TCF7L2_F and TCF7L2_R. (a) CC homozygote for rs7903146 at 63rd position of the PCR product, (b) CT heterozygote, and (c) TT homozygote. PCR: Polymerase chain reaction; SNP: Single nucleotide polymorphism.

For the comparison of the accuracy of the allele-specific polymerase chain reaction (AS-PCR) method used for the study, we have randomly chosen the samples that were sent for sequencing. The result of the sequencing was a 100% match to the finding of the AS-PCR genotyping method (Figure 3).

## Discussion

In recent times diabetes in India has become a pandemic. This may be attributed to the shift in environmental or lifestyle factors and susceptibility to hereditary genes which predisposes an individual to T2DM. One of the major associated genes to T2DM reported by Grant et al. in their linkage study was TCF7L2 [6]. The study indicated the susceptibility of variants in rs7903146, rs12255372, and DG10S478 microsatellite. In the present study, rs7903146 SNP and its association with T2DM is investigated.

TCF7L2 gene belongs to the family of high mobility group box-containing transcription factors involved in the Wnt signaling pathway. The SNP rs 7903146 is located on chromosome 10q25.3 in the 4th intron of the TCF7L2 gene. The Wnt signaling pathway is involved in the regulation of cell proliferation, cell differentiation, polarity determination, and tissue homeostasis during embryonic development [8].

Activation of the Wnt signaling pathway takes place when a Wnt ligand binds to a Frizzled family transmembrane receptor on the cell. This is followed by an intracellular signaling cascade that leads to the inactivation of a β-catenin destruction complex [9]. β-catenin is spared from destruction and eventually, it moves from the cytoplasm to the nucleus. In the nucleus, it interacts with TCF7L2 protein and other transcription factors. There it removes transcriptional repression and helps in recruiting coactivators for inducing target genes [10]. TCF7L2 gene plays an important role in the development of pancreatic islet and β-cell function via Wnt signaling. TCF7L2 protein induces the expression of various genes, including the insulin tropic hormone glucagon-like peptide-1 (GLP-1) gene, the insulin gene, and other genes that encode proteins involved in the processing and exocytosis of insulin granules [11]. Any alteration in transcriptional regulation of target genes due to variation in the TCF7L2 gene leads to β-cell dysfunction, which is the main etiology of T2DM.

The present study was conducted on 194 T2DM patients recruited from the endocrinology department at Indira Gandhi Institute of Medical Sciences, Patna, and 180 non-diabetic healthy controls. In the diabetic group HbA1c, and plasma glucose (fasting, post-prandial, and random) were increased significantly. This was expected in diabetes, the occurrence of hyperglycemia is caused by deficient production of insulin and insulin resistance. In the same diabetic subjects LDL, VLDL, total cholesterol, and triglycerides were also increased highly significantly and HDL cholesterol was decreased significantly compared to the control group. The cause of dyslipidemia in the diabetic group may be attributed to insulin resistance which exerts its effect via different mechanisms. Insulin resistance is associated with reduced inhibition of hormone-sensitive lipase in adipose tissue, thereby augmenting portal flux of free fatty acid (FFA). FFA and triglycerides derived from FFA or FFA inhibit apolipoprotein B (apoB) degradation in the liver, thereby stimulating the overproduction of VLDL1 and is metabolically associated with a preponderance of small dense LDL and reduced large cholesterol-rich HDL2 [12].

Our study aimed to investigate the potential association of TCF7L2 rs7903146 polymorphism in T2DM patients. We performed AS-PCR on the extracted DNA from the subjects. The genotype frequency observed in diabetic patients, the wild-type CC, CT, and TT genotypes were 24.2%, 39.3%, and 16.5% respectively. On the other hand, 38.9% of the controls had the CC genotype, 48.3% had the CT genotype, and 12.8% had the TT genotype. There were statistically significant differences observed between the cases and controls regarding the genotype frequencies (p< 0.05). C allele was present in 53.9% of the cases and 63.1% of the controls (p<0.05). The T allele was found in 46.1% of the cases as compared to 36.9% of the controls (p< 0.05). This result shows the preponderance of the T-risk allele in the diabetic group.

There was almost a two-fold risk associated with the mutant homozygous TT genotype as compared to the wild-type CC genotype. A similar risk of 1.96 times was associated with heterozygote CT compared to CC.

Our results were in agreement with studies done on the Indian population and other ethnicity around the world. Chandak et al. have found a 1.46 times higher risk of diabetes in the south Indian population with the rs 7903146 SNP mutant allele [13]. Uma et al. have found a strong association of TCF7L2 gene polymorphisms with T2DM in the population of Hyderabad [14]. In the western Indian population, Chauhan et al. reported a 67% higher risk associated with T2DM having TT homozygous for the SNP [15]. Sanghera et al. and Bodhini et al. have also reported similar findings in their study on the north Indian Khatri and the south Indian population respectively [16,17].

Ding et al. in their meta-analysis did not find significant heterogeneity in Caucasian, East Asian, South Asian, and other ethnicity [18]. They have reported a significant association of rs7903146 with T2DM in all the above populations [18]. A study on T2DM patients from the Khyber Pakhtunkhwa population showed a significant risk of T allele either in heterozygote or homozygous state [19]. Similar studies on African, Asian, and Brazilian subjects have reported a strong association of the SNP to T2DM [20].

Although we have not looked for the association of dyslipidemia and rs7903146 polymorphism in the present study, one study by López-Ortiz et al. has reported that there is no association of minor T allele and plasma TC, LDL-cholesterol, or HDL-cholesterol levels in subjects with T2DM or metabolic syndrome [21]. When they fed the nopal tortilla diet to subjects with CC genotype they found elevated GLP-1 levels. The GLP-1 increases insulin secretion and favors satiety, which may in turn cause loss of weight [21].

TCF7L2 rs7903146 is also associated with type I diabetes mellitus and a singlet islet autoantibody positivity supporting that non-autoimmune pathways are involved in a subset of autoimmune type I diabetes [22].

While most of the studies around the world are in agreement with our study some studies are not in consensus with the present study. Li Zhu et al. in their study of the Han Chinese population reported no association of rs7903146 SNP with T2DM, this was not in concordance with other studies and meta-analyses on the Han Chinese population [23,24,25]. Not a significant amount of literature has refuted the association of rs7903146 SNP with T2DM in the Indian population.

The mechanism by which TCF7L2 gene single nucleotide variations (SNV) increase susceptibility to T2DM is still not clear, also rs7903146 SNP is located in the intron. A question that needs to be investigated is how the intronic variants affect TCF7L2 gene expression.

Cropano et al. have reported that TCF7L2 rs7903146 is associated with hepatic but not peripheral insulin resistance in diabetes [26].

Oh et al. have shown in their mice model the influence of insulin resistance on the expression of TCF7L2 in the liver, which contributes to increased glucose production and resultant hyperglycemia [27].

Studies by Lyssenko et al. suggested the effect of rs7903146 of TCF7L2 T allele on the enteroinsular axis and the relationship between the incretin hormone GIP and its target hormones, glucagon and insulin [4]. They also found increased expression of TCF7L2 associated with T allele in human islets in vitro and impaired insulin secretion both in vitro and in vivo.

Some studies have indicated the implication of chromatin state in variants and their role in pathology. Gaulton et al. reported that in human islets, the chromatin state at the TCF7L2 locus is more open in chromosomes carrying the rs7903146 T allele. To this date, we do not know the exact mechanism of such a chromatin state in the causation of T2DM [28].

## Conclusions

Our study shows that the rs7903146 (C/T) polymorphism is associated with T2DM in the eastern Indian population. However, our findings may be confirmed by further studies with larger sample sizes. Also, the future therapeutic application of diagnosis of the SNP may be explored in the treatment of diabetes such as response to drug and treatment modality. Screening for the polymorphism may help prevent the onset of T2DM or in delaying the effects of T2DM.
